# Proof-of-concept study for a long-acting formulation of ivermectin injected in cattle as a complementary malaria vector control tool

**DOI:** 10.1186/s13071-022-05621-z

**Published:** 2023-02-14

**Authors:** Sié Hermann Pooda, Nicolas Moiroux, Angélique Porciani, Anne-Laure Courjaud, Christophe Roberge, Georges Gaudriault, Issa Sidibé, Adrien Marie Gaston Belem, Jean-Baptiste Rayaissé, Roch K. Dabiré, Karine Mouline

**Affiliations:** 1 Université de Dédougou, Dedougou, Burkina Faso; 2 Centre International de Recherche et Développement pour l’Élevage en zones Sub-humides, Bobo-Dioulasso, Burkina Faso; 3 Insectarium de Bobo Dioulasso – Campagne d’éradication de la mouche Tsé Tsé et des Trypanosomoses, Bobo-Dioulasso, Burkina Faso; 4 MIVEGEC, Université de Montpellier-CNRS-IRD, Montpellier, France; 5 Institut de Recherche en Sciences de la Santé, Bobo Dioulasso, Burkina Faso; 6 MedinCell S.A., Jacou, France; 7 Université Nazi Boni, Bobo-Dioulasso, Burkina Faso

**Keywords:** *Anopheles*, Malaria, Residual transmission, Ivermectin, Long-acting formulation, Cattle, One health, Burkina Faso

## Abstract

**Background:**

Domesticated animals play a role in maintaining residual transmission of *Plasmodium* parasites of humans, by offering alternative blood meal sources for malaria vectors to survive on. However, the blood of animals treated with veterinary formulations of the anti-helminthic drug ivermectin can have an insecticidal effect on adult malaria vector mosquitoes. This study therefore assessed the effects of treating cattle with long-acting injectable formulations of ivermectin on the survival of an important malaria vector species, to determine whether it has potential as a complementary vector control measure.

**Methods:**

Eight head of a local breed of cattle were randomly assigned to either one of two treatment arms (2 × 2 cattle injected with one of two long-acting formulations of ivermectin with the BEPO^®^ technology at the therapeutic dose of 1.2 mg/kg), or one of two control arms (2 × 2 cattle injected with the vehicles of the formulations). The lethality of the formulations was evaluated on 3–5-day-old *Anopheles coluzzii* mosquitoes through direct skin-feeding assays, from 1 to 210 days after treatment. The efficacy of each formulation was evaluated and compared using Cox proportional hazards survival models, Kaplan–Meier survival estimates, and log-logistic regression on cumulative mortality.

**Results:**

Both formulations released mosquitocidal concentrations of ivermectin until 210 days post-treatment (hazard ratio > 1). The treatments significantly reduced mosquito survival, with average median survival time of 4–5 days post-feeding. The lethal concentrations to kill 50% of the *Anopheles* (LC_50_) before they became infectious (10 days after an infectious blood meal) were maintained for 210 days post-injection for both formulations.

**Conclusions:**

This long-lasting formulation of ivermectin injected in cattle could complement insecticide-treated nets by suppressing field populations of zoophagic mosquitoes that are responsible, at least in part, for residual malaria transmission. The impact of this approach will of course depend on the field epidemiological context. Complementary studies will be necessary to characterize ivermectin withdrawal times and potential environmental toxicity.

**Graphical Abstract:**

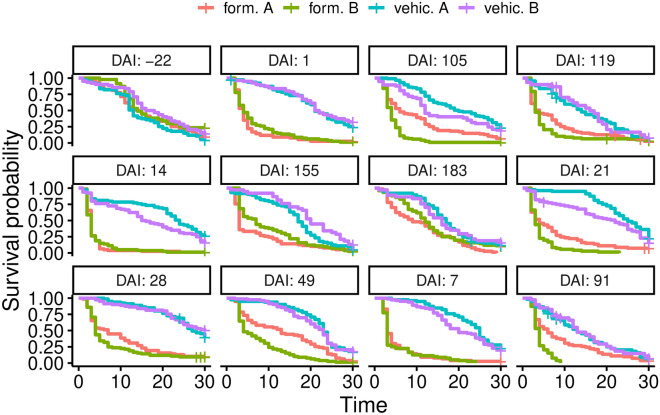

**Supplementary Information:**

The online version contains supplementary material available at 10.1186/s13071-022-05621-z.

## Introduction

From 2000 to 2015, an estimated 663 million malaria cases are thought to have been averted in the world [1]. Among these, 68% may be attributed to the use of long-lasting insecticidal nets (LLINs) and 13% to residual insecticide spraying. As such, vector control remains by far the most effective approach to control disease transmission. Despite this progress, malaria is an ongoing serious public health concern worldwide and this disease still impairs the social and economic development of endemic countries. In 2020, there were an estimated 241 million new cases of malaria and 627,000 deaths [2]. These figures represent an increase of approximately 10% in the number of deaths compared to 2019, even though three quarters of these deaths may be attributable to the COVID-19 crisis and the saturation of health systems [2]. The fact remains that in recent years, malaria incidence and mortality have increased in several African regions.

This increase raises the issue of the limits of current prevention approaches. The vector control side faces limited access to LLINs, as well as the resistance of malaria mosquitoes to four out of the five classes of insecticides approved for malaria vector control, namely, to pyrethroids, carbamates, organophosphates, and organochlorides [2–4]. Resistance can occur through mutations rendering the insecticide target site insensitive to the molecule, through increased metabolic detoxification processes, or through structural adaptations that mitigate the effect of the insecticide [5]. Aside from this physiological resistance, additional limits come from behavioral features that primary and secondary vectors display. These behaviors, such as exophagy, zoophagy, incongruous patterns of biting, or a co-association between some if not all of these traits, allow the mosquitoes to overcome control tools and to maintain or increase parasite transmission [6–9]. Hence, the control of residual malaria will necessarily require targeted abatement of the populations of these particular vectors [9].

The World Health Organization (WHO) Global Technical Strategy for Malaria Control 2016–2030 requires the development of innovative vector control tools that can be integrated into contemporary malaria control programs. The use of endectocides, and in particular ivermectin, is considered an additional option to target vector mosquito populations and residual transmission [10, 11].

Ivermectin is a broad-spectrum anthelminthic medication that was first licensed in 1981 for veterinary use [12]. Since 1987, it has been approved for human use and widely distributed through mass drug administration (MDA) campaigns all over Africa to eliminate onchocerciasis, lymphatic filariasis, and other parasitic diseases [13]. Studies have shown that ivermectin is lethal for hematophagous vector species, including malaria vector mosquitoes, when they absorb their blood meal from ivermectin-treated humans or animals [14]. Ivermectin has proven mosquitocidal properties for all the malaria vector species tested to date [15–17] and as such, the drug has triggered interest in malaria transmission control. It should therefore be possible to suppress entire vector populations, in a similar manner to the community-level impacts of LLINs [9, 18, 19] by administering ivermectin to hosts in order to poison mosquitoes when they feed upon them. This new approach offers several advantages.

1. There is a high probability that vector mosquitoes feeding (even just once) on hosts treated with ivermectin will die before becoming infectious, i.e., before the end of the extrinsic incubation period of the *Plasmodium* parasite, ≈10 days.
2. The drug’s mode of action is different from all the other insecticides currently used in vector control. As such, malaria mosquitoes that are physiologically resistant to pyrethroids and other insecticide classes used for LLINs can be effectively targeted to mitigate ongoing insecticide resistance problems. In addition, ivermectin targets only invertebrate-specific glutamate-gated chloride channels. It also binds to a P-glycoprotein membrane efflux pump [20] corresponding to a multidrug-resistant glycoprotein that prevents the molecule from crossing the blood/brain barrier [21]. As such, for animals, including humans, ivermectin has an excellent safety profile even at doses higher than recommended [22].
3. The drug should be effective for all vector mosquito species regardless of their behaviors: the period when they are aggressive, the location where they prefer to bite (indoors or outdoors), or their feeding preferences (zoophagic vectors could also be targeted). Hence, it offers the potential for extending insecticide coverage beyond humans to also encompass livestock and the residual transmission they feed. This falls into the “One Health” context, where both animal and human health are improved.
4. Sublethal concentrations of ivermectin impair fecundity, fertility, and mobility of *Anopheles* mosquitoes [23] meaning that even mosquitoes that survive after exposure to ivermectin are impaired in their reproductive fitness, which further contributes to vectors populations suppression.

More than four billion doses have been administered en masse to humans since 1987 by the Mectizan Donation Program, with the aim of eliminating lymphatic filariasis and onchocerciasis [24]. Adverse severe effects with therapeutic doses have been registered, but at an extremely low rate over many years of monitoring, and are usually associated with high parasite infection loads, particularly in the presence of *Loa loa* co-infection [21, 22, 25, 26].

The systemic insecticide potential of ivermectin administered to humans or animals has been thoroughly demonstrated in the laboratory (e.g., [13, 15], reviewed in [11, 16]), in small-scale field trials [27, 28], and in different ecological settings in the field where a decrease of the sporozoite prevalence in the vector population has been demonstrated following the MDA of ivermectin to humans [29, 30]. In addition, Foy et al. conducted the first randomized control trial in Burkina Faso to demonstrate the epidemiological impact of ivermectin on malaria [31]. The authors showed that in addition to the use of LLINs, repeated mass administration of single doses of ivermectin every 3 weeks during the rainy season could reduce malaria incidence by 20% in children aged 5 or under ([31], but see [32, 33]).

Despite its high potential for malaria control, the use of ivermectin poses several challenges. A single dose is not enough to maintain the lethal concentrations required to decrease vector populations for a duration that would significantly impact *Plasmodium* transmission. Ivermectin cannot as yet impact transmission because of the relatively short plasma half-life of market formulations (about 18–56 h in humans [22], and from 24 to 364 h in cattle, depending on the formulation used [34]). When administered subcutaneously to cattle, the classical veterinary formulation of ivermectin renders animals’ blood toxic to *Anopheles* for up to 3 weeks post-injection [17].

Using higher doses and/or repeated doses of the current formulations of ivermectin could be a way to enhance and sustain its impact. However, an MDA strategy based on the use of most current formulations would suffer from strong limitations. Given the short half-life of the drug in humans or animals, repeated administrations would be needed, which would be extremely challenging with respect to logistics, costs, and sustained compliance of the populations.

Another approach could consist in developing new formulations allowing sustained plasmatic concentrations above the LC_50_ for a longer period. WHO recognizes this challenge and suggests developing a formulation releasing ivermectin for at least a month, preferably during most of the rainy season [10].

For this purpose, technologies allowing the slow, long-lasting release of a drug would increase the *Plasmodium* transmission control potential and aid in repurposing of ivermectin administration for malaria vector control. As such, experimental long-lasting implants releasing mosquitocidal concentrations of ivermectin for 6 months have been developed and tested in rabbits [35] and cattle [36]. Such a device is inserted and retrieved by minor surgical procedures, and dose adjustments to animals’ weight made through the number of implants. Like Chaccour’s, our work followed from the expressed need for a mosquitocidal product with a release duration spanning most of a transmission season. To achieve this, we used an in situ depot technology (BEPO^®^, [37]) and designed an ivermectin long-lasting formulation that we believe could be easier to manage in the field context than an inserted implant. The design is based on the use of block copolymers that entrap the therapeutic molecule upon depot solidification when in contact with body fluids. The depot progressively bioresorbs while delivering the active pharmaceutical ingredient with the desired pharmacokinetics. This technology was previously used to develop an ivermectin formulation which was tested for its microfilaricidal effect against *Onchocerca ochengi* [38].

The objective of the present study is to evaluate the malaria vector control potential of an alternative long-acting injectable formulation designed to release ivermectin for 6 months at lethal concentrations for *Anopheles coluzzii*, one of the major malaria vectors in sub-Saharan Africa. Mosquitocidal effects and efficacy duration were tested by injecting the formulation in cattle at the classical veterinary therapeutic dose of 0.2 mg/kg/month. Experiments were performed in laboratory settings of Burkina Faso, where we treated local cattle bread and, at different time points spanning the formulation’s expected efficacy duration of 6 months, we performed direct skin-feeding assays for survival follow-up using an *An. coluzzii* colony.

## Methods

### Mosquito colony

We used a colony of *An. coluzzii* mosquitoes from the Burkinabe Institut de Recherche en Sciences de la Santé du Burkina Faso, Direction Régionale de l’Ouest (IRSS DRO) established in 2008 using gravid females collected in Bama, Kou Valley (11° 23′ 14″ N, 4° 24′ 42″ W), 30 km from Bobo-Dioulasso, in southwestern Burkina Faso (West Africa). To alleviate founder effects and to maintain representative genetic diversity, the colony is replenished every year with first filial generation (F1) progeny from at least 50 mosquito females caught in the same locality, after the species status is identified by routine polymerase chain reaction (PCR) [39]. Potential contamination of the colony by other *Anopheles* species is routinely checked using the same technique.

The mosquito colony was maintained under the following standard conditions: a temperature of 27 ± 2 °C, relative humidity of 75 ± 5%, and a 12 h/12 h day/night cycle. Larvae were reared in plastic trays containing tap water and fed ad libitum with commercial alevin food (TetraMin^®^ Baby). Pupae were collected in cups and placed in cages measuring 30 × 30 × 30 cm. Newly emerged adults were allowed to feed for 3–5 days on a 5% glucose solution and then starved for 16–18 h before blood-feeding on cattle.

### Cattle hosts

Eight bull calves of the local Metis breed (obtained by the cross-breeding of Fulani zebus and Baoulé cattle) were used as hosts for *Anopheles* direct skin-feeding assays. This study was carried out in accordance with the ethical guidelines for the care of laboratory animals (Act no. 00468, 24 January 1994) applicable in all West African French-speaking countries.

Upon their arrival at the cowshed of the Centre International de Recherche-Développement sur l’Élevage en zone Subhumide (CIRDES), the calves were treated with therapeutic doses of aceturate diminazene and albendazole to cure potential trypanosomiasis (endemic in this area) and gastrointestinal infestation with endoparasites, respectively. To our knowledge, no study has ever reported an effect of these molecules on *Anopheles* survival or fecundity but the calves were nevertheless left to acclimatize and clear these drugs in the cowshed for a month before the start of our experiment, during which time they were protected by a net to avoid any ectoparasite disturbance and reinfestation. Calves were fed a diet consisting of straw and cotton oil cake, and provided with water and salt ad libitum. They were checked every 2 days by a veterinarian to ensure their well-being. The weight gain of the calves served as an indicator of their well-being: the calves were weighed before the start of the experiment and after its completion to determine the percentage of mass change over the course of the experiment.

### Manufacturing of slow-release formulations of ivermectin

The alternative injectable long-acting formulations of ivermectin were designed using the BEPO^®^ technology [37] and prepared as described in [38]. Briefly, a triblock copolymer, PLA_97_–PEG_45_–PLA_97_, and two diblock copolymers, mPEG_45_–PLA_130_ and mPEG_7_–PLA_41_, were synthesized by ring-opening polymerization in bulk condition as already described (patent US 9023897 B2). Two long-lasting formulations were synthesized: (1) formulation A, composed of 5% (w/w) of ivermectin, 45% (w/w) of copolymer comprising PLA_97_–PEG_45_–PLA_97_ and mPEG_45_–PLA_130_ and 50% (w/w) DMSO (dimethyl sulfoxide); and (2) formulation B, composed of 5% (w/w) ivermectin, 50% (w/w) of a copolymer comprising PLA_97_–PEG_45_–PLA_97_ and mPEG_7_–PLA_41_ and 45% (w/w) DMSO. Before preparation, the tri- and di-block copolymers of each formulation were first dissolved overnight in DMSO (Procipient, Gaylord Chemical), at room temperature, and under continuous stirring. ivermectin (Fagron, France) was then added to the polymer solution until its complete dissolution. The formulations were sterile filtered using 0.2 µm filters (Minisart SRP 15, Sartorius) and then administered to cattle according to their body mass (1.2 mg of ivermectin/kg of body mass, i.e., 24 mg of formulation/kg), using a hypodermic syringe capped with a 16-gauge needle.

Long-lasting formulations of ivermectin were imported into Burkina Faso under the clearance provided by the Direction Générale des Services Vétérinaires of Burkina Faso (Permit No. 14/107 issued on 18 November 2014).

### Treatment of calves

Calves were randomly assigned to receive either the vehicles (i.e., the copolymers dissolved in DMSO, without ivermectin) or the treatments (ivermectin-containing formulations). The formulations were injected subcutaneously into the loose skin in front of the shoulder. Calf number C8 moved suddenly during the injection, resulting in the withdrawal of the needle before the end of the injection. Hence, for this calf, the injection was made in two different places instead of only one. Two calves (C1 and C6) were randomly assigned to each of the four arms: vehicle A: C2 and C4, treatment A: C1 and C3, vehicle B: C5, C7, treatment B: C6, C8.

### Blood-feeding

For each blood-feeding assay, 3- to 5-day-old female mosquitoes from the same batches of eggs were randomly introduced into 32 plastic cups covered with nets (*n* = 50–70 mosquitoes per cup) 16–18 h before the direct skin blood-feeding. Mosquitoes were starved; only a cotton ball soaked with water was left in the cup to increase their propensity to feed on the hosts. Four plastic cups were randomly assigned to each calf, placed on the animal’s sides, and held with a rubber strap tied around the abdomen. Animals were carefully restrained with ropes to avoid rough movements or scratching. Mosquitoes were allowed to feed for 15 min, after which only fully engorged females were transferred in cardboard cups for survival follow-ups. Blood-feeding of mosquitoes occurred at 14 different points in time during the experiment: 22 days before treatment, and after the injections on days 1, 7, 14, 21, 28, 49, 91, 105, 119, 155, 183, 195, and 210. The percentage of blood-fed mosquitoes (90–95%) was similar between the 14 batches, for each treatment and each calf (data not shown).

### Ivermectin bioanalysis

After the blood meals (except on days 105 and 119 after injection because of a logistical failure) and for each calf, 5 ml of blood was withdrawn from the jugular vein in heparinized tubes (BD Vacutainer^®^ PST™ tubes). Blood samples were centrifuged at 2500×*g* for 10 min at room temperature. Next, 1.5 ml of the supernatant (i.e., the blood plasma) was transferred in plastic tubes and stored at −20 °C until further processing. Samples were analyzed for their ivermectin content as described by Boussinesq et al. [38].

### Evaluation of the survival of mosquitoes fed on treated and control cattle

Fully engorged females were randomly distributed and maintained in paper cups for the survival follow-up. Four cups were used per calf with 10 mosquitoes per cup and provided every day with cotton balls soaked in a 2.5% glucose solution. Mortality was recorded every day from the day of the blood meal and for 30 days afterward. Mosquitoes that were alive on the 30th day were registered as “censored.” In total, 3378 mosquitoes were observed daily for 30 days.

### Statistical analysis and modeling

All statistical analyses and modeling were performed using the software RStudio version 4.0.1 [40]. The data and the R codes are all available upon request.

### Statistical analysis

#### Dynamics of ivermectin in cattle blood

Ivermectin concentration dynamics as a function of time were characterized for each formulation and caw by fitting generalized additive models (GAM) with a cubic regression spline to compare formulations A and B and assess the potential variation in the pharmacodynamics of ivermectin between individual cattle.

#### Mosquito survival

Kaplan–Meier survival estimates were calculated to investigate if the longevity of females, estimated during a follow-up of 30 days, was affected by a blood meal taken on treated cattle on different days after injection [0–210 days after injection (DAI)] and for each formulation and vehicles. The effects of the vehicle, the formulations, the time elapsed since injection, and their interactions on survival were further tested using Cox proportional hazards mixed models with cattle as nested random effects. Additional survival analysis with Cox models and Kaplan–Meier estimates were performed to characterize potential confounding effects due to variations between individual cattle blood before the ivermectin formulations were injected (0 DAI).

Complementary analyses were carried out in the framework of the efficacy study to consider the concentrations of ivermectin that were found in treated calves, at different points in time (as in Fig. 3, except for DAI = 105 and 119 where ivermectin concentration was not available). A mixed-effects Cox regression model was used to characterize the impacts of the formulations, ivermectin concentration, and their interaction on mosquito survival. Variance associated with point in time since injection (DAI) and identities of individual cattle were considered as random effects.

**Fig. 1:**
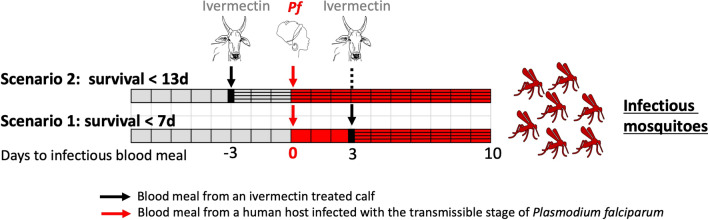
Schematic representation of the two blood-feeding scenarios considered in this study, in which an *Anopheles* mosquito feeds in an area where cattle are mass-treated with a long-lasting formulation of ivermectin A or B. *Pf*: *Plasmodium falciparum*. In gray: the mosquito does not carry *Pf*. In red: the mosquito carries *Pf* and eventually becomes infectious. Hatched areas represent for each scenario, the time during which the mosquito should die after its ivermectin blood meal to ensure that the transmission of sporozoites does not occur

We further explored the efficacy of the two formulations by considering the probability that a mosquito ingesting a blood meal containing ivermectin dies before it becomes infected with *Plasmodium falciparum* sporozoites. We considered two simple scenarios: (1) the female mosquito ingests a blood meal containing ivermectin after it ingested an infectious blood meal (2) the female mosquito ingests a blood meal containing ivermectin before ingesting an infectious blood meal. We assumed a gonotrophic cycle of 3 days, and 10 days as the average number of days required by *Plasmodium* to undergo sporogony, as in other studies using ivermectin [41]. We examined the effect of the formulations on cumulative mortality 7 and 13 days (*n* = 7, *n* = 13) after the ivermectin blood meal (see Fig. 1 for a schematic representation of both scenarios). Data were considered as binomial [i.e., dead (1) or not dead (0) before *t* = *n* days after ivermectin blood-feeding]. Dose–effect of ivermectin concentration on mosquitoes' survival until a given day post-treatment (7 or 13 days) was assessed using a four-parameter dose–response log-logistic regression for different times of mortality during a follow-up of 30 days (see Additional file 1: Tables S1 and S2).

When needed, analyses were followed by post hoc tests to compare the levels of significant factors.

## Results

### Formulations, ivermectin concentration, and stable drug release

**Fig. 2:**
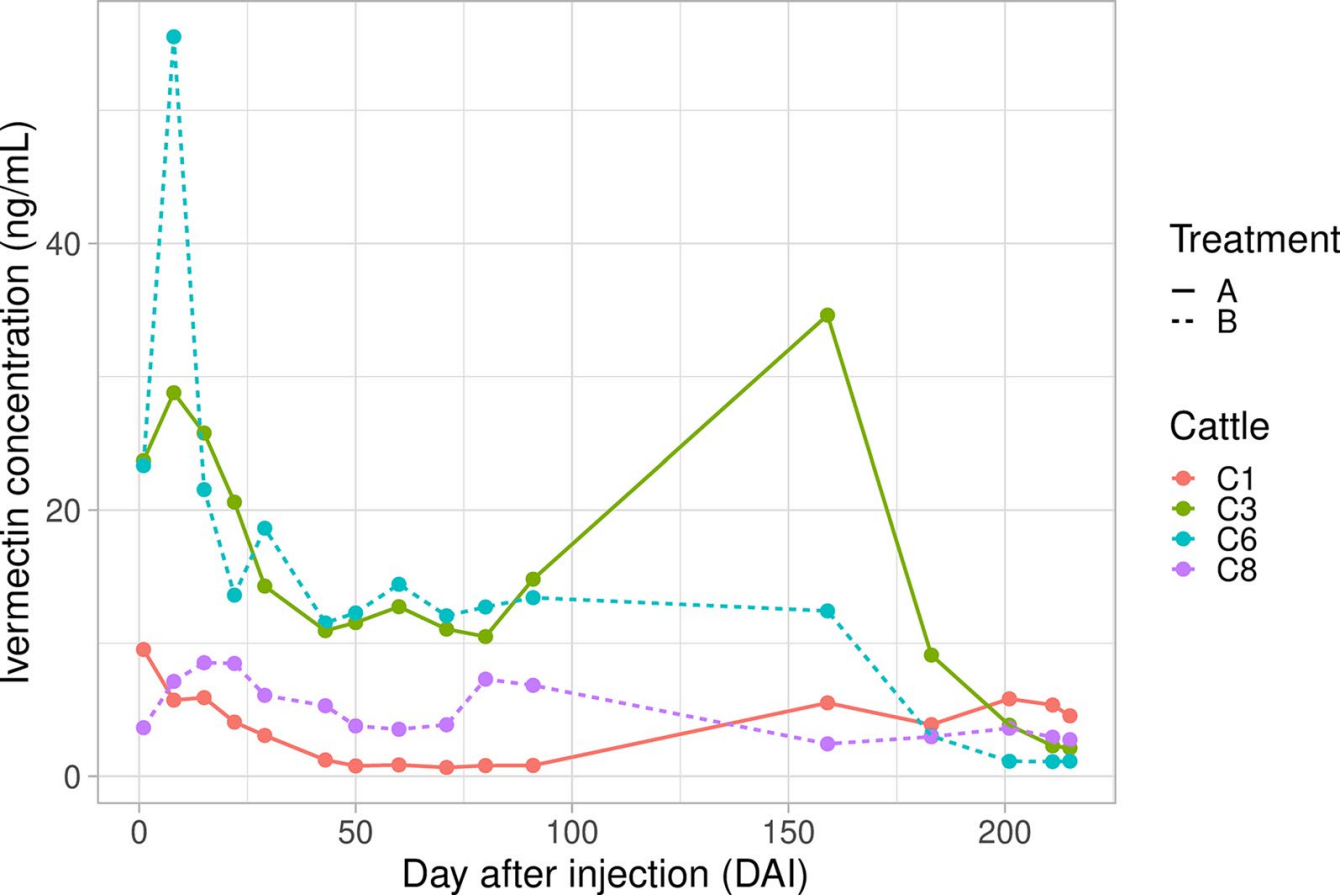
Ivermectin plasma concentrations (ng/ml) measured over time in calves after injection of the long-acting ivermectin formulations A and B. Calves C1 and C3 received formulation A while C6 and C8 received formulation B

Comparison of the two ivermectin treatment arms reveals that the composition of the long-acting formulations (i.e., the polymer mixture which traps the ivermectin) did not yield different plasma concentrations of the molecule (formulation effect, *F* = 0.062, *P* = 0.8). For each formulation, the ivermectin plasma concentration profile varies over time with more or less fluctuation depending on the calf (Fig. 2). Significant fluctuations in the concentration–time profile are observed for the calves C3 (treated with formulation A) and C6 (treated with B) (i.e., a significant time effect, C3: *F* = 16.58, *P* < 0.001; C6: *F* = 22.867, *P* < 0.001), while for the other calves, the concentration remains steady [time effect, C1(FA): *F* = 1.551, *P* = 0.278; C8(FB): *F* = 1.612, *P* = 0.211]. For both C3 and C6, ivermectin concentrations show an initial burst whose highest values reach more than 40 ng/ml 7 days after injection and remain most of the time above 10 ng/ml. It is worth noting that for these calves, plasma concentrations dramatically decrease between days 165 and 185, which corresponds to the targeted duration of release. After day 185, ivermectin concentrations continue to decrease until they reach values close to the limit of quantification (0.1 ng/ml). For C1 and C8, the plasma-concentration–time profile unexpectedly looks different with an absence of an initial burst release and relatively steady concentrations of ivermectin in the range of 2–10 ng/ml from day 0 to 210.

### Efficacy study: survival of mosquitoes over time after skin-feeding assays

**Fig. 3:**
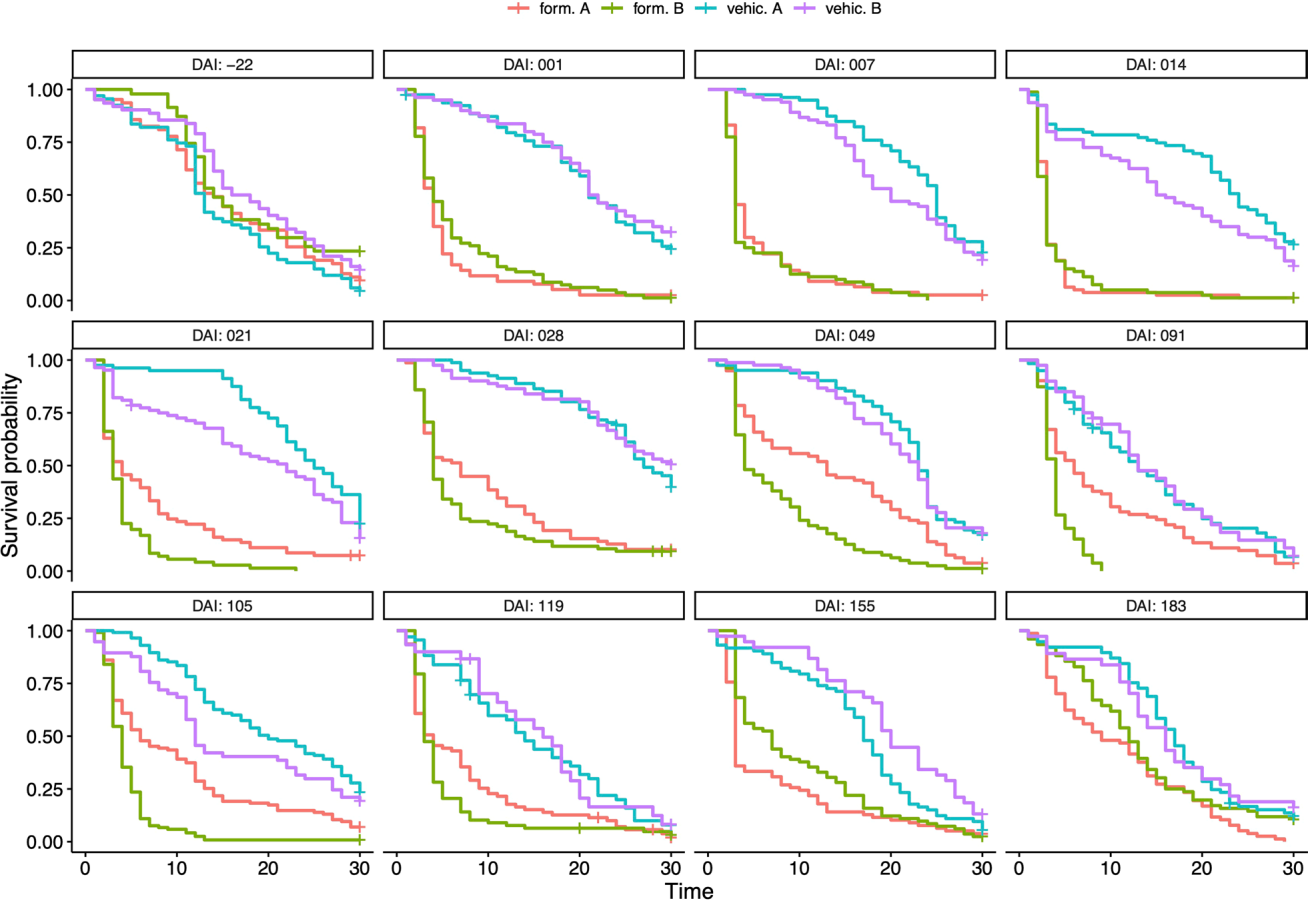
Kaplan–Meier survival curves for mosquitoes which fed on the calves of each experimental arm. Curves are drawn for each period of time elapsed after injection, i.e., the day on which mosquitoes were fed during direct skin-feeding experiments. *DAI* days after injection

Before treatment, no survival difference was observed between the four experimental groups (host identity and cage number were both considered as random effects, likelihood ratio test (LRT) *χ*_3_^2^ = 0.68, *P* = 0.88, Fig. 3, additional file 2 DAI = −22).

Kaplan–Meyer survival curves were drawn for each group after treatment (Fig. 3) to illustrate the treatment effect for each formulation and at each time point after injection. Multivariate survival analysis showed a significant effect of the days elapsed after injection (DAI), the treatment, and their interaction, upon mosquito survival (DAI effect: *χ*_10_^2^ = 192.05, *P* < 0.001; treatment effect: *χ*_3_^2^ = 27.42, *P* < 0.001; DAI × treatment effect: *χ*_30_^2^ = 289.67, *P* < 0.001), meaning that the survival probability estimated for each DAI depends on the treatment.

When compared, formulations A and B showed the same overall level of efficacy during the experiment (see Fig. 3 and Additional file 3: Table S3). The effect of formulation A compared to the control was not significant at two specific points in time (*t* = 49 and *t* = 91 days after injection, Fig. 3). Also, at the end of the follow-up, i.e., 180 days post-treatment (which is the targeted product profile), the toxic effect of both formulations upon mosquitoes is not significant anymore.

When considering efficacy as a function of the ivermectin concentration, the Cox model shows that there is a significant effect of ivermectin concentration (LRT*χ*_1_^2^ = 184.57, *P* < 0.001), formulations (LRT*χ*_1_^2^ = 6.57, *P* = 0.01), and their interaction (LRT*χ*_1_^2^ = 22.86, *P* < 0.001) on mosquito survival, which suggests that even for the same ivermectin plasma concentration, the efficacy of formulations A and B are apparently not the same.

**Fig. 4:**
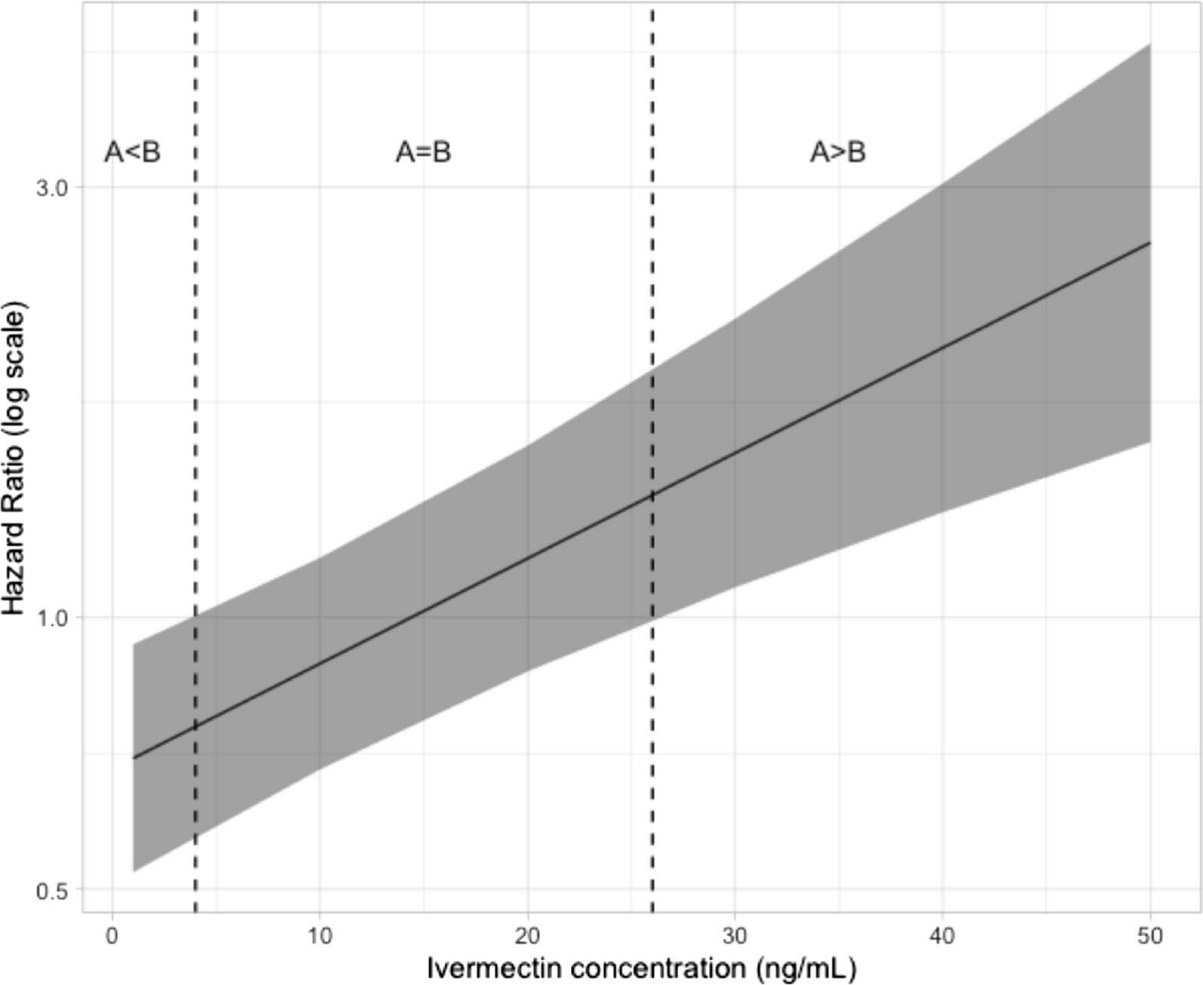
Estimated hazard ratio (HR) as a function of ivermectin concentration for comparison of formulation A and B efficacy at killing mosquitoes. Gray ribbon represents 95% confidence interval of HR estimation

The Cox model predicted an increase in the probability of mosquito death when the concentration of ivermectin was increased in the range of 1–50 ng/ml (*P* < 0.001). For each additional 1 ng/ml, the model estimated that the daily mortality rate should be multiplied by 1.08 for formulation A, and by 1.05 for formulation B. For lower ivermectin concentrations in plasma (< 4 ng/ml), the probability of death was estimated to be better if formulation B was used (hazard ratio [HR] < 1), whereas formulation A seemed more effective (HR > 1) at higher concentrations (> 26 ng/ml) (Fig. 4).

**Fig. 5:**
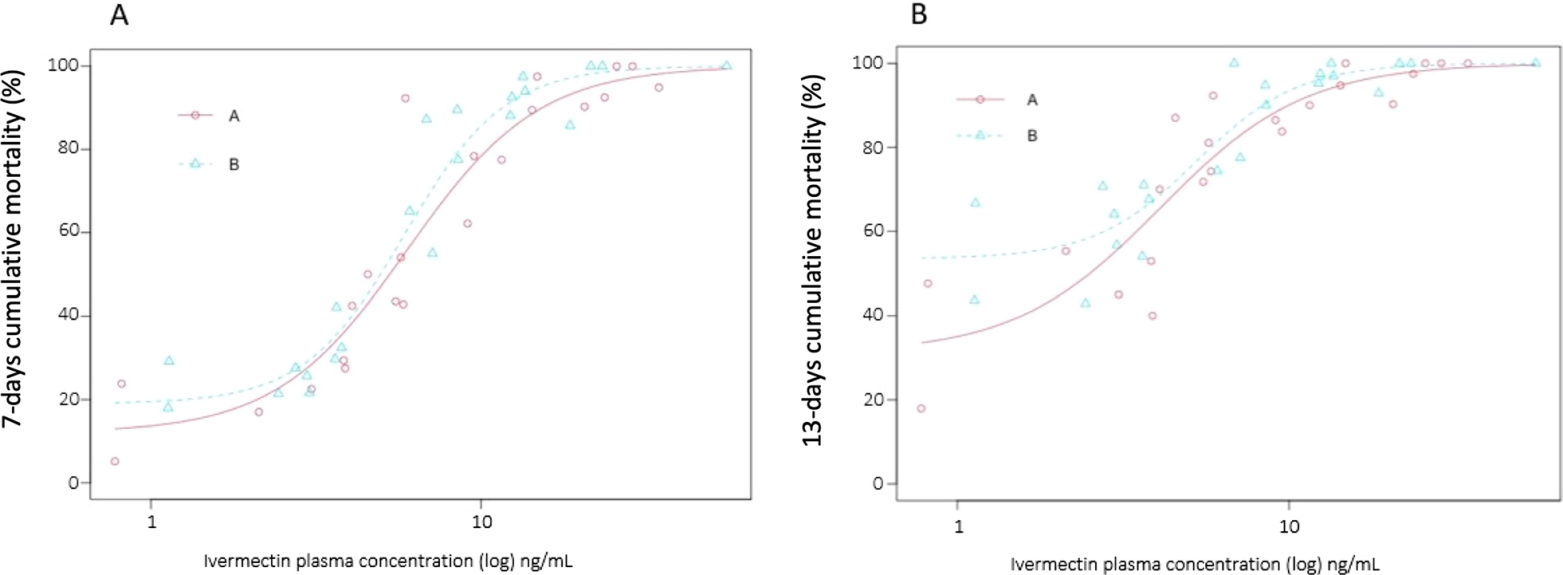
Estimation of the dose–response relationship between ivermectin plasma concentrations and mosquito mortality when calves are injected with formulation A (solid red lines, red circles) or B (dotted blue lines, blue triangles). Lines are the estimated relationships following the log-logistic regression, while circles and triangles represent means of experimental data. Ivermectin-induced mortality is explored according to two scenarios A and B that would decrease the parasites’ transmission: **A** the mosquito dies within 7 days after an ivermectin blood meal (ivermectin blood meal is taken after the infectious one); **B** the mosquito dies within 13 days after the ivermectin blood meal (ivermectin blood meal is taken before the infectious one). Details of the two scenarios are given in the methods section and Fig. 1

When considering epidemiologically relevant mosquito survival times (Fig. 1), the dose–response regression predicts that formulation B would induce a mosquito mortality rate of 90% before *Plasmodium* has time to undergo sporogony if ivermectin plasma concentrations reached 12.56 ng/ml 95% CI (10.78; 14.34) and 11.46 ng/ml 95% CI (9.25; 13.67) ng/ml, for scenarios 1 and 2, respectively. Formulation A would induce a mortality rate of 90% at higher concentrations, 16.4 ng/ml 95% CI (13.59; 19.22) for scenario 1 and 12.32 ng/ml 95% CI (19.69; 14.96) ng/ml for scenario 2. Furthermore, the LC_50_ values estimated for scenario 1 (50% of dead mosquitoes before 7 days post-ivermectin blood meal) are 5.38 ng/ml 95% CI (4.89; 5.87) and 6.08 ng/ml 95% CI (5.5; 6.66) for formulations B and A, respectively. For scenario 2, LC_50_ are 5.26 ng/ml 95% CI (3.94; 6.6) and 4.09 ng/ml 95% CI (3.05; 5.13) for formulations B and A (Fig. 5).

## Discussion

Our study was designed to establish the proof of concept that a long-lasting formulation of ivermectin could allow the release, in a single injection, of mosquitocidal plasma concentrations of ivermectin to kill malaria vector mosquitoes for up to 6 months. This sustained effect and the associated logistical advantages for mass administration to domesticated animals reinforce the perspectives for the One Health approach and the fight against malaria in different field contexts [42].

For the two tested formulations, intra- and inter-animal variability in the pharmacokinetic profiles were observed. Fluctuation in ivermectin concentration has also been observed in other studies [38]. In addition, physiological factors, such as body mass index, unique to each individual animal, may carry some degree of variability. The body fat in cattle, where ivermectin is accumulated, could act as a reservoir from which ivermectin is released in the function of each individual metabolism, which differs between hosts [43]. Such a hypothesis was raised by Ouédraogo et al. [41] to explain ivermectin concentration differences between treated male and female humans. In the same idea, species-specific basal metabolic rates explain differences in ivermectin concentration profiles over time and its efficacy against parasites [34]. Recently, Tipthara et al. [44] showed that ivermectin metabolism by humans produces compounds that may extend the insecticidal action of the core molecule. Hence, metabolite differences, either quantitative or qualitative, are likely to be found as well between species and from one individual to another, and may impact efficacy.

We showed that for specific ranges of ivermectin concentrations, identical amounts seem to induce different mosquito mortality rates depending on which formulation is used. This was not expected, and could be due to the formulation composition or to the injection process. Moreover, even with the random effect integrated in the model, such difference should be taken with caution because outcomes might be influenced by the limited number of calves that we used per experimental arm. This is a true limit for characterizing the overall profile of ivermectin release, but not to conclude that such formulation has great potential for malaria vector control. Further studies to test long-acting ivermectin formulations, incorporating more replicate animals per group, are definitely needed to understand as accurately as possible the pharmacokinetic profiles and efficacy variability according to the formulation composition and dosage.

A previous study attempted to use the same technology to fight natural infections of zebus by *O. ochengi* [38]. It allowed a year-round release of microfilaricidal ivermectin concentrations. Both formulations in our study reach these concentrations as well, and as such the use of long-lasting formulations could have additional benefits and support the fight against other endo- or ectoparasitic diseases of animals, including zoonoses that are transmitted to humans [45].

With plasma concentrations reaching at least the LC_50_ for *Anopheles* species for 6 months, our formulations have the potential to help circumvent the technical and logistical gaps identified by WHO for the use of ivermectin for mass administration to humans or animals [10], For a One Health approach, administering ivermectin to animals is not expected to be beneficial in all field contexts. Suitability scores computed by Imbahale et al. [46] identified the Sahelian zone as the region which would benefit the most from this approach, especially due to the zoophagic behavior of *Anopheles arabiensis*, one of the main *Plasmodium* vectors of this area.

Among all the formulations that have been tested so far, ours offers the unique advantage of being injected in a single shot and of progressive resorption while releasing active concentrations of ivermectin for a duration that could largely encompass the *Plasmodium* transmission season. The LC_50_ values found in our study were in the same range as previous in vivo experiments reports [41, 47, 48], and are compatible with the therapeutic effects of ivermectin for the treatment of parasitic diseases in humans and animals [47]. For these reasons, the concept of an injectable long-acting formulation of ivermectin appears to be an effective tool to combat malaria transmission through a mass treatment of cattle, and ultimately of all domesticated animals that are hosts for *Anopheles* vectors. Indeed, multiple dosing programs seem hardly compatible with field logistical requirements.

Whatever the formulation, administering ivermectin to animals will require further studies to consider the risk of ivermectin resistance in the populations of helminths that are classically treated using ivermectin [49], but also, in *Anopheles* populations [48]. The selection pressure exerted by ivermectin will increase dramatically if the approach is deployed on a mass scale, and the emergence of ivermectin resistance would likely be only a matter of time, as has been the case for all other insecticidal compounds widely used to date. Ideally, mitigation strategies based on careful monitoring of existing or future markers should be proposed and implemented together with the approach, in all targeted field contexts. The threat of selecting the most anthropophagous mosquitoes by targeting domesticated animals and zoophagic behaviors has to be assessed as well. Moreover, mitigation strategies should also explore the environmental toxicity issues that would ensue with the release of ivermectin in the excrement of treated hosts. These could have potentially dramatic consequences on non-targeted terrestrial or aquatic fauna, especially for numerous species of dung-degrading insects that are crucial for soil fertilization [50]. In addition, ivermectin has been reported to be phytotoxic as well [51]. Today, the need for environmentally sustainable solutions must be borne in mind, even if ivermectin seems to be a promising solution to increase the health of both humans and animals by killing malaria mosquitoes and parasites responsible for zoonotic human and livestock diseases that impair local development. Ongoing studies on already marketed ivermectin formulations attempt to measure the ivermectin amounts released in the feces of treated cattle to evaluate toxicity on reference non-targeted coprophagic species. These studies should give initial findings on the associated risks in the fields [52]. Treatments and mitigation measures should be defined and developed with the help and expertise of local herders and peasants, which would support their full participation in the overall approach.

Concentration studies will also have to be conducted to determine the ivermectin withdrawal period in the different edible cattle tissues and organs, and in milk. A 6-month release period from a long-lasting ivermectin formulation may indeed lead to qualitatively and quantitatively different accumulation of the compound that could necessitate increased withdrawal time when compared, for example, to the recommended 28 days for classical products for cattle [53]. This should be characterized by taking into account the recommendations and frameworks of the Joint Food and Agriculture Organization (FAO)/WHO Expert Committee on Food Additives (JECFA, [54, 55]). Basically, gestating and lactating females and animals intended for human or animal meat or milk consumption during the ivermectin releasing period should not be considered, unless owners' will and non-consumption guarantees are given. Males dedicated to labor and young animals will on the contrary be primarily targeted. Cattle herders and owners’ involvement will be more than necessary to define and adapt the injections coverage according to their needs and requirements, and modeling approaches will help in defining the most efficient co-constructed treatment scheme.

Aside from *Plasmodium*-bearing mosquitoes, other insects of medical and veterinary importance are sensitive to ivermectin as well [45]. Novel ivermectin formulations like the one in our study could serve as broader control tools by targeting other neglected tropical diseases of zoonotic origin. In theory, this innovative tool could definitely be integrated within a One Health approach, with benefits for both animals and humans. Because resistance risks are potent, the use of other endectocides is a possible mitigation strategy to address the appearance of ivermectin resistance in both helminths and *Anopheles*.

## Conclusion

Our study shows that a formulation releasing mosquitocidal concentrations of ivermectin for 6 months could provide a complementary malaria control measure. This tool could be effective in endemic areas where domesticated animals live in close vicinity to human beings, both in urban and rural environments. More studies are needed to further assess inter-individual variability and tune the pharmacokinetic profile of ivermectin to the optimal characteristics. To this end, future animal studies will be sufficiently powered to afford more robust data interpretation and projections in the fields. Treating animals using a long-lasting formulation of ivermectin within a One Health paradigm, combined with a currently deployed antimalarial arsenal, would surely help impacting harder the malaria transmission and incidence, in the ranges expected by WHO.

## Supplementary Information

**Additional file 1:** More details on the methodology.

**Additional file 2.** Four-parameter log-logistic model equation.

**Additional file 3: Table S3**. Hazard ratio value of multiple comparisons.

## Data Availability

All data generated or analyzed during this study are included in this published article (and its supplementary information files).

## References

[1] Bhatt S, Weiss DJ, Cameron E, Bisanzio D, Mappin B, Dalrymple U, Battle KE, Moyes CL, Henry A, Eckhoff PA, Wenger EA, Briët O, Penny MA, Smith TA, Bennett A, Yukich J, Eisele TP, Griffin JT, Fergus CA, Lynch M, Lindgren F, Cohen JM, Murray CLJ, Smith DL, Hay SI, Cibulskis RE, Gething PW (2015). The effect of malaria control on *Plasmodium falciparum* in Africa between 2000 and 2015. Nature.

[2] World malaria report 2021 [Internet]. [cited 2022 July 13]. https://www.who.int/teams/global-malaria-programme/reports/world-malaria-report-2021.

[3] Ranson H, N’Guessan R, Lines J, Moiroux N, Nkuni Z, Corbel V (2011). Pyrethroid resistance in African anopheline mosquitoes: what are the implications for malaria control?. Trends Parasitol.

[4] Ranson H, Lissenden N (2016). Insecticide resistance in African anopheles mosquitoes: a worsening situation that needs urgent action to maintain malaria control. Trends Parasitol.

[5] Corbel V, N’Guessan R. Distribution, mechanisms, impact and management of insecticide resistance in malaria vectors: a pragmatic review [Internet]. Anopheles mosquitoes—new insights into malaria vectors. IntechOpen; 2013 [cited 2022 Jan 6]. https://www.intechopen.com/chapters/43899.

[6] Carrasco D, Lefèvre T, Moiroux N, Pennetier C, Chandre F, Cohuet A (2019). Behavioural adaptations of mosquito vectors to insecticide control. Curr Opin Insect Sci.

[7] Moiroux N, Gomez MB, Pennetier C, Elanga E, Djenontin A, Chandre F, Djègbé I, Guis H, Corbel V (2012). Changes in *Anopheles funestus* biting behavior following universal coverage of long-lasting insecticidal nets in Benin. J Infect Dis.

[8] Moiroux N, Damien GB, Egrot M, Djenontin A, Chandre F, Corbel V, Killeen GF, Pennetier C (2014). Human exposure to early morning *Anopheles funestus* biting behavior and personal protection provided by long-lasting insecticidal nets. PLoS ONE.

[9] Killeen GF, Kiware SS, Okumu FO, Sinka ME, Moyes CL, Massey NC, Gething PW, Marshall JM, Chaccour CJ, Tusting LS (2017). Going beyond personal protection against mosquito bites to eliminate malaria transmission: population suppression of malaria vectors that exploit both human and animal blood. BMJ Glob Health.

[10] Endectocide and ectocide products for malaria transmission control [Internet]. [cited 2022 July 12]. https://www.who.int/publications/i/item/9789240052512.

[11] Roadmappers TI (2020). A roadmap for the development of ivermectin as a complementary malaria vector control tool. Am J Trop Med Hyg.

[12] Campbell J, Kessler B, Mayack C, Naug D (2010). Behavioural fever in infected honeybees: parasitic manipulation or coincidental benefit?. Parasitology.

[13] Molyneux DH, Bradley M, Hoerauf A, Kyelem D, Taylor MJ (2003). Mass drug treatment for lymphatic filariasis and onchocerciasis. Trends Parasitol.

[14] Strong L, Brown TA (1987). Avermectins in insect control and biology: a review. Bull Entomol Res.

[15] Chaccour C, Lines J, Whitty CJ (2010). Effect of ivermectin on *Anopheles gambiae* mosquitoes fed on humans: the potential of oral insecticides in malaria control. J Infect Dis.

[16] Chaccour CJ, Kobylinski KC, Bassat Q, Bousema T, Drakeley C, Alonso P, Foy BD (2013). Ivermectin to reduce malaria transmission: a research agenda for a promising new tool for elimination. Malar J.

[17] Pooda HS, Rayaisse JB, Hien DF, Lefevre T, Yerbanga SR, Bengaly Z, Dabiré RK, Belem AM, Sidibé I, Solano P, Mouline K (2014). Administration of ivermectin to peridomestic cattle: a promising approach to target the residual transmission of human malaria. Malar J.

[18] Hawley WA, Phillips-Howard PA, Kuile FOT, Terlouw DJ, Vulule JM, Ombok M, Nahlen BL, Gimnig JE, Kariuki SK, Kolczak MS, Hightower AW (2003). Community-wide effects of permethrin-treated bed nets on child mortality and malaria morbidity in western Kenya. Am J Trop Med Hyg.

[19] Killeen GF, Smith TA, Ferguson HM, Mshinda H, Abdulla S, Lengeler C, Kachur SP (2007). Preventing childhood malaria in Africa by protecting adults from mosquitoes with insecticide-treated nets. PLoS Med.

[20] Cordon-Cardo C, O’Brien JP, Casals D, Rittman-Grauer L, Biedler JL, Melamed MR, Bertino JR (1989). Multidrug-resistance gene (P-glycoprotein) is expressed by endothelial cells at blood-brain barrier sites. Proc Natl Acad Sci.

[21] Schinkel AH, Smit JJM, van Tellingen O, Beijnen JH, Wagenaar E, van Deemter L, Mol CA, Van der Valk MA, Robanus-Maandag EC, Te Riele HP, Berns AJ (1994). Disruption of the mouse mdr1a P-glycoprotein gene leads to a deficiency in the blood-brain barrier and to increased sensitivity to drugs. Cell.

[22] Guzzo CA, Furtek CI, Porras AG, Chen C, Tipping R, Clineschmidt CM, Sciberras DG, Hsieh JY, Lasseter KC (2002). Safety, tolerability, and pharmacokinetics of escalating high doses of ivermectin in healthy adult subjects. J Clin Pharmacol.

[23] Sampaio VS, Beltran TP, Kobylinski KC, Melo GC, Lima JB, Silva SG, Rodriguez ÍC, Silveira H, Guerra MG, Bassat Q, Pimenta PF (2016). Filling gaps on ivermectin knowledge: effects on the survival and reproduction of Anopheles aquasalis, a Latin American malaria vector. Malar J.

[24] 35 Years: The Mectizan® Donation Program [Internet]. Merck.com. [cited 2022 July 12]. https://www.merck.com/stories/mectizan/.

[25] Boussinesq M, Kamgno J, Pion SD, Gardon J (2006). What are the mechanisms associated with post-ivermectin serious adverse events?. Trends Parasitol.

[26] Chandler RE (2018). Serious neurological adverse events after ivermectin—do they occur beyond the indication of onchocerciasis?. Am J Trop Med Hyg.

[27] Sylla M, Kobylinski KC, Gray M, Chapman PL, Sarr MD, Rasgon JL, Foy BD (2010). Mass drug administration of ivermectin in south-eastern Senegal reduces the survivorship of wild-caught, blood fed malaria vectors. Malar J.

[28] Lyimo IN, Kessy ST, Mbina KF, Daraja AA, Mnyone LL (2017). Ivermectin-treated cattle reduces blood digestion, egg production and survival of a free-living population of *Anopheles arabiensis* under semi-field condition in south-eastern Tanzania. Malar J.

[29] Kobylinski KC, Sylla M, Chapman PL, Sarr MD, Foy BD (2011). Ivermectin mass drug administration to humans disrupts malaria parasite transmission in Senegalese villages. Am J Trop Med Hyg.

[30] Alout H, Krajacich BJ, Meyers JI, Grubaugh ND, Brackney DE, Kobylinski KC, Diclaro JW, Bolay FK, Fakoli LS, Diabaté A, Dabiré RK (2014). Evaluation of ivermectin mass drug administration for malaria transmission control across different West African environments. Malar J.

[31] Foy BD, Alout H, Seaman JA, Rao S, Magalhaes T, Wade M, Parikh S, Soma DD, Sagna AB, Fournet F, Slater HC (2019). Efficacy and risk of harms of repeat ivermectin mass drug administrations for control of malaria (RIMDAMAL): a cluster-randomised trial. Lancet Lond Engl.

[32] Bradley J, Moulton LH, Hayes R (2019). Analysis of the RIMDAMAL trial. Lancet.

[33] Foy BD, Rao S, Parikh S, Slater HC, Dabiré RK (2019). Analysis of the RIMDAMAL trial—authors’ reply. Lancet.

[34] Canga A, Sahagún Prieto AM, Diez M, Fernandez N, Sierra M, Garcia J (2009). The pharmacokinetics and metabolism of ivermectin in domestic animal species. Vet J.

[35] Chaccour C, Barrio A, Royo A, Urbistondo D, Slater H, Hammann F, Del Pozo JL (2015). Screening for an ivermectin slow-release formulation suitable for malaria vector control. Malar J.

[36] Chaccour CJ, Ngha’bi K, Abizanda G, Irigoyen Barrio A, Aldaz A, Okumu F, Slater H, Del Pozo JL, Killeen G (2018). Targeting cattle for malaria elimination: marked reduction of *Anopheles arabiensis* survival for over six months using a slow-release ivermectin implant formulation. Parasit Vectors.

[37] Roberge C, Cros J-M, Serindoux J, Cagnon M-E, Samuel R, Vrlinic T, Berto P, Rech A, Richard J, Lopez-Noriega A (2020). BEPO®: bioresorbable diblock mPEG-PDLLA and triblock PDLLA-PEG-PDLLA based in situ forming depots with flexible drug delivery kinetics modulation. J Control Release.

[38] Boussinesq M, Enyong P, Chounna-Ndongmo P, Njouendou A-J, Pion SD, Rech A, Roberge C, Gaudriault G, Wanji S (2020). Effects of an injectable long-acting formulation of ivermectin on Onchocerca ochengi in zebu cattle. Parasite.

[39] Santolamazza F, Mancini E, Simard F, Qi Y, Tudella Torre ZA (2008). Insertion polymorphisms of SINE200 retrotransposons within speciation islands of Anopheles gambiae molecular forms. Malar J.

[40] R: The R Project for Statistical Computing [Internet]. [cited 2022 July 12]. https://www.r-project.org/.

[41] Ouedraogo AL, Bastiaens GJ, Tiono AB, Guelbeogo WM, Kobylinski KC, Ouedraogo A, Barry A, Bougouma EC, Nebie I, Ouattara MS, Lanke KH (2015). Efficacy and safety of the mosquitocidal drug ivermectin to prevent malaria transmission after treatment: a double-blind, randomized, clinical trial. Clin Infect Dis.

[42] Chaccour C (2021). Veterinary endectocides for malaria control and elimination: prospects and challenges. Philos Trans R Soc B Biol Sci.

[43] Baraka OZ, Mahmoud BM, Marschke CK, Geary TG, Homeida MM, Williams JF (1996). Ivermectin distribution in the plasma and tissues of patients infected with *Onchocerca volvulus*. Eur J Clin Pharmacol.

[44] Tipthara P, Kobylinski KC, Godejohann M, Hanboonkunupakarn B, Roth A, Adams JH, White NJ, Jittamala P, Day NP, Tarning J (2021). Identification of the metabolites of ivermectin in humans. Pharmacol Res Perspect.

[45] Laing R, Gillan V, Devaney E (2017). Ivermectin—old drug, new tricks?. Trends Parasitol.

[46] Imbahale SS, Montaña Lopez J, Brew J, Paaijmans K, Rist C, Chaccour C (2019). Mapping the potential use of endectocide-treated cattle to reduce malaria transmission. Sci Rep.

[47] Smit MR, Ochomo EO, Aljayyoussi G, Kwambai TK, Abong’o BO, Chen T, Bousema T, Slater HC, Waterhouse D, Bayoh NM, Gimnig JE (2018). Safety and mosquitocidal efficacy of high-dose ivermectin when co-administered with dihydroartemisinin-piperaquine in Kenyan adults with uncomplicated malaria (IVERMAL): a randomised, double-blind, placebo-controlled trial. Lancet Infect Dis.

[48] Nicolas P, Kiuru C, Wagah MG, Muturi M, Duthaler U, Hammann F, Mai M, Chaccour C (2021). Potential metabolic resistance mechanisms to ivermectin in Anopheles gambiae: a synergist bioassay study. Parasit Vectors.

[49] Sutherland IA, Leathwick DM (2011). Anthelmintic resistance in nematode parasites of cattle: a global issue?. Trends Parasitol.

[50] Verdu JR, Cortez V, Ortiz AJ, Gonzalez-Rodriguez E, Martinez-Pinna J, Lumaret JP, Lobo JM, Numa C, Sánchez-Piñero F (2015). Low doses of ivermectin cause sensory and locomotor disorders in dung beetles. Sci Rep.

[51] Vokřál I, Michaela Š, Radka P, Jiří L, Lukáš P, Dominika S, Kateřina L, Barbora S, Lenka S (2019). Ivermectin environmental impact: excretion profile in sheep and phytotoxic effect in Sinapis alba. Ecotoxicol Environ Saf.

[52] Ivermectin against malaria: a one-health approach to treat humans and peridomestic animals with regard to minimal ecological side-effects [Internet]. Agence Natl. Rech. [cited 2022 July 12]. https://anr.fr/Project-ANR-17-CE35-0013.

[53] Slanina P, Kuivinen J, Ohlsén C, Ekström LG (1989). Ivermectin residues in the edible tissues of swine and cattle: effect of cooking and toxicological evaluation. Food Addit Contam.

[54] JECFA|Food safety and quality| Food and Agriculture Organization of the United Nations [Internet]. [cited 2022 Nov 22]. https://www.fao.org/food-safety/scientific-advice/jecfa/en/.

[55] Canton L, Lanusse C, Moreno L (2021). Rational pharmacotherapy in infectious diseases: issues related to drug residues in edible animal tissues. Animals.

