# Supplementary material for: Proof-of-concept study for a long-acting formulation of ivermectin injected in cattle as a complementary malaria vector control tool
Source: Parasit Vectors. 2023 Feb 14;16:66. doi: 10.1186/s13071-022-05621-z (PMC9926456; doi:10.1186/s13071-022-05621-z)
Supplement: Supplementary file 1 — Additional file 1: More details on the methodology. [file 13071_2022_5621_MOESM1_ESM.pdf]

Additional file 1

Dynamic of IVM in cattle blood:

The IVM concentration in the plasma of the four treated bovine was fitted to the number of day post injection using a Generalized Additive Model (GAM) with automatic choice of smoothing parameters. We used the “gam” function of the “mgcv” package (1) in “R” (2). The GAM model was then used to predict the mean concentration of IVM in bovine plasma from 1 to 250 days post-injection.

Effect of IVM on Anopheles mortality:

We assume that a vector biting on a given day after host IVM injection will experience an increased risk of death and, moreover, it will die at a new constant mortality rate governed by the amount of IVM in the cattle blood at the time the blood meal was taken. The GAM model was used to estimate this amount of IVM. A Cox proportional hazards survival model was used to describe how this concentration of IVM affects mortality. The relationship between the log of the concentration of IVM ingested by the mosquito and its mortality was modeled using a second-order polynomial function. The hazard from the survival model was converted into a mortality rate by multiplying the baseline mortality rate (0.1) by the relevant hazard for mosquitoes biting on each day post host IVM injection. We used the “coxph” function of the “survival” package (3) in “R” for this task.

Malaria transmission model:

Susceptible-Exposed-Infectious (SEI) model of *P. falciparum* transmission

The model described by Slater *et al.* (2014) (4) was modified to account for two types of host (cattle and human) and for the effect on vector mortality of two interventions (IVM in cattle and LLINs in humans).

State variables and parameters of the model are described in Supplementary Table 2.

We defined the time that IVM is injected to cattle as s. The length of time that IVM has a mosquitocidal effect is denoted l and is taken as the number of days that the mortality rate is greater than the baseline mortality rate ($\mu_{v0} = 0.1$). We then define an ‘on-off’ switch to determine whether IVM is having an effect of the vector population of not.

Each day a proportion of mosquito blood meal is taken on cattle (*1-HBI*) of which a proportion (based on the IVM coverage rate C_{ivm}) will contain IVM. These vectors then move to a new compartment where they will have a higher mortality rate based on the amount of time after IVM injection that the bloodmeal was taken. Once vectors moved to the new compartment on the specified day they will have that mortality rate for the rest of their lifespan. The mortality rate for vector biting on day i post IVM injection is denoted μ_v^{di} and is given by the vector mortality model (Figure 3). The mortality rate of all non-IVM ingested mosquitoes equals the baseline mortality rate μ_{v0}.

Each day a proportion of susceptible mosquitoes take a blood meal on humans (*HBI*). A proportion of these vectors will move to the infected compartment based on the *P. falciparum* prevalence (P_{pf}) in the human population and on an infectiousness probability (k). Infected vectors will move to the infectious compartment after n days (duration of the extrinsic incubation of *P. falciparum*).

Among the vectors that take a blood meal on humans, a proportion μ_h will die due to the presence of LLINs.

Supplementary Table S1: State variables and parameters of the Susceptible-Exposed-Infectious (SEI) model of *P. falciparum* transmission

State variables and parameters	Definition	Value
S_v	Susceptible vectors that have not taken a bloodmeal containing IVM	-
E_v	Exposed vectors that have not taken a bloodmeal containing IVM	-
I_v	Infectious vectors that have not taken a bloodmeal containing IVM	-
S_v^{di}	Susceptible vectors that took a bloodmeal containing IVM on day i post-injection	-
E_v^{di}	Exposed vectors that took a bloodmeal containing IVM on day i post- injection	-
I_v^{di}	Infectious vectors that took a bloodmeal containing IVM on day i post-injection	-
P_{pf}	*Plasmodium falciparum* prevalence in the human population	0.5
k	Infectiousness: probability that a vector become infectious while taking a blood meal on an infectious host	0.1
g	Duration of the gonotrophiq cycle	2
BR	Biting rate : rate at which a vector take a blood meal (in days^{-1})	1/g
n	Duration of *P. falciparum* extrinsic incubation	11
s	Day of IVM injection	50
l	Duration of IVM efficacy (in days)	250
E	New vector births per day	300
μ_{v0}	Baseline daily mortality (The mortality rate of all non-IVM ingested mosquitoes)	0.1
μ_v^{di}	Daily mortality rate for vector biting on day i post IVM injection	see Figure 3
μ_h	Probability of death due to LLIN when encountering a human.	Vector behavior model eq. (2)
HBI	Human Blood Index: Proportion of blood meal taken from humans	Vector behavior model eq. (3)
C_{ivm}	Proportion of cattle treated with IVM	0 or 1
p_{ivm}	Probability that a vector take a bloodmeal on an IVM treated cattle	$(1 - HBI) \times C_{ivm}$
p_{inf}	Probability that a fed vector become infectious	$k \times P_{pf} \times HBI$

Using the following function to move mosquitoes to a new compartment:

$$\delta(t,\tau,d) = \begin{cases} 1 \; if \; \tau \leq t < \tau + d \\ 0 \quad \text{otherwise} \end{cases}$$

The following equations are used to describe the system:

Susceptible, no IVM

$$\frac{dS_v}{dt} = E - \mu_{v0} Sv - \left(\mu_h HBI + p_{inf}\,(1 - \mu_h) + \delta(t,s,l) p_{IVM}\right) BR\, Sv$$

Susceptible, IVM (for i = 1,…,l)

$$\frac{dS_v^{di}}{dt} = \delta(t,s,l)p_{IVM}BR\,Sv\ -\ \mu_v^{di}S_v^{di} - (p_{inf}\,(1\ -\ \mu_h) +\ \mu_h HBI)BR\,S_v^{di}$$

Exposed, no IVM

$$\frac{dE_v}{dt} = p_{inf}\,(1\ -\ \mu_h)S_v - \left(\mu_{v0} + \frac{1}{n}\right)E_v - (\delta(t,s,l)p_{IVM} +\ \mu_h HBI\,)BR\,E_v$$

Exposed, IVM (for i = 1,…, ,l)

$$\frac{dE_v^{di}}{dt} = \delta(t,s,l)p_{IVM}E_v + p_{inf}\,(1\ -\ \mu_h)BR\,S_v^{di} - \left(\mu_v^{di} + \frac{1}{n} +\ \mu_h HBI\,BR\right)E_v^{di}$$

Infectious, no IVM

$$\frac{dI_v}{dt} = \frac{1}{n}\,E_v -\ \mu_{v0}I_v - (\delta(t,s,l)p_{IVM} +\ \mu_h HBI)\,BR\,I_v$$

Infectious, IVM (for i = 1,…,l)

$$\frac{dI_v^{di}}{dt} =\ \delta(t,s,l)p_{IVM}\,BR\,I_v + \frac{1}{n}E_v^{di} - (\mu_v^{di} +\ \mu_h HBI\,BR)\,I_v^{di}$$

Vector behavior model:

A vector behavior model was developed to feed the SEI model of *P. falciparum* transmission with credible values of (i) probability that a vector will feed on cattle (1 - *HBI*) and (ii) probability of death due to the LLINs μ_h (for vectors encountering a human) under various environmental and entomological scenario:

- varied cattle:human ratio in the host population (i.e. more cattle, equal number of human and cattle, or more humans),

- varied levels of LLIN coverage in the human host population (0, 50 or 100%) and,

- varied host preference phenotype (human *vs.* cattle) in the *Anopheles* population (zoophilic, opportunistic or anthropophilic).

We assume that the probability at which a vector chooses a type of host (human or cattle) is independent on the origin of a previous blood meal and that LLIN have no remote effect (i.e. no deterrence). Parameters of the vector behavior model are described in Supplementary Table 3 .

Supplemental table S2: Parameters used in the vector behavior model

Parameter	Definition	Value	Source
C_h	Net coverage : proportion of the human population that use LLINs	Varied (0; 0.5; 1)	-
$r_{C:H}$	Cattle:human ratio	Varied (0.5; 1; 2)	-
a	Preference for human (against cattle) as it would be measured in a dual choice olfactometer	Varied (0.2; 0.5; 0.8)	-
P_{ii}	Proportion of exposure to bite during which LLIN is in use	0.9	(5,6)
$\mu_{h.u}$	Death probability when encountering an unprotected host in an experimental hut (EH)	0.0485	(7), data for Permanet2 in the Kou Valley area.
$\mu_{h.p}$	Death probability when encountering a human protected by an LLIN in an EH	0.4438	
f_{hu}	Successful feeding probability when encountering an unprotected human in an EH	0.7566	
f_{hp}	Successful feeding probability when encountering a human protected by an LLIN in an EH	0.3526	

Calculation of the probability of death due to the LLINs (for a vector encountering a human)

We assume that malaria vector can take a blood meal either on human or cattle. Human are considered either protected if they use LLINs or unprotected. The probability for a vector that an encountering human is protected by an LLIN equal the proportion of human P_p that are protected by an LLIN. Giving the LLIN coverage (C_h) in the human population and the proportion of exposure to bite during which LLIN is in use (P_{ii}), we calculated P_p as follow:

$$P_p = C_h \times P_{ii} \qquad (1)$$

The use of LLINs in the human population increase the probability of mortality for vectors encountering human hosts. From experimental hut trial (EHT) data, we can calculated $\mu_{h.p}$, the probability of death for a vector entering a hut with a human protected by an LLIN, and $\mu_{h.u}$, the probability of death for one entering a hut (i.e. the control hut) with an unprotected human. The difference between $\mu_{h.p}$ and $\mu_{h.u}$ is the mortality induced by the presence of the LLIN. Knowing the proportion of human P_p that are protected by an LLIN (equation (1)), we can deduce the probability of death due to the LLIN (μ_h) when encountering a human as follow:

$$\mu_h = (\mu_{h.p} - \mu_{h.u}) \times P_p \qquad (2)$$

Calculation of the probability that a vector fed on cattle

Each day, the proportion of fed vectors having taken a blood meal on human is the realized Human blood index (*HBI*). It can be express as:

$$HBI = F_h / (F_h + F_c) \qquad (3)$$

With F_h, the probability that, on one night, a vectors successfully feed on human and F_c, the probability that a vector successfully feeds on cattle.

F_h and F_c are the product of, for each host, its relative availability in the community (A), the probability of successful feeding (f) when encountering this host and the preference (a) of the vector population for this host:

$F_h = A_h \times f_h \times a$ (4)

$F_c = A_c \times f_c \times (1 - a)$ (5)

The preference (a) for human hosts is defined as the intrinsic preference (i.e. phenotypic) of the vector population, i.e. the proportion of a sample of the vector population that would choose the human host in a dual-choice (human vs. cattle odor) olfactometer.

Relative availability of each host can be expressed as the proportion it represent in the total host population. Giving a defined ratio of cattle per human ($r_{C:H}$) in a community and the proportion P_p (equation (1)) of human that are protected by an LLIN, we can calculated :

The proportion A_h of host that are humans:

$A_h = 1 / (1 + r_{C:H})$ (6)

The proportion A_p of hosts that are protected human:

$A_p = A_h \times P_p$ (7)

The proportion A_u of hosts that are unprotected human:

$A_u = A_h \times (1 - P_p)$ (8)

And the proportion A_c of hosts that are cattle:

$A_c = 1 – A_h$ (5)

Successful feeding probabilities when encountering a protected (f_{hp}) or unprotected human (f_{hu}) were deduced from EHT data by dividing the number of blood fed vector collected in the huts by the total number having entered the huts (for both treated and control huts, respectively). Successful feeding probability when encountering a cattle was considered equal to the one of unprotected human. We therefore can calculate F_h and F_c as follow:

$F_h = (A_p \times f_{hp} + A_u \times f_{hu}) \times a$ (6)

$F_c = A_c \times f_{hu} \times (1 - a)$ (7)

F_h and F_c can then be used to calculate *HBI* by solving equation (3).

The probability that a fed vector took its blood meal on a calf is therefore equal to *1 – HBI.*

References

1. Wood SN. Fast stable restricted maximum likelihood and marginal likelihood estimation of semiparametric generalized linear models. J R Stat Soc Ser B Stat Methodol. 2011 Jan 1;73(1):3–36. doi:10.1111/j.1467-9868.2010.00749.x

2. R Development Core Team. R: A Language and Environment for Statistical Computing [Internet]. Vienna, Austria: R Foundation for Statistical Computing; 2010. Available from: http://www.R-project.org

3. Therneau TM. A Package for Survival Analysis in S [Internet]. 2015. Available from: https://CRAN.R-project.org/package=survival

4. Slater HC, Walker PGT, Bousema T, Okell LC, Ghani AC. The potential impact of adding ivermectin to a mass treatment intervention to reduce malaria transmission: a modelling study. J Infect Dis. 2014 Dec 15;210(12):1972–80. doi:10.1093/infdis/jiu351

5. Huho B, Briet O, Seyoum A, Sikaala C, Bayoh N, Gimnig J, et al. Consistently high estimates for the proportion of human exposure to malaria vector populations occurring indoors in rural Africa. Int J Epidemiol. 2013;42(1):235–47. doi:10.1093/ije/dys214

6. Moiroux N, Damien GB, Egrot M, Djenontin A, Chandre F, Corbel V, et al. Human exposure to early morning Anopheles funestus biting behavior and personal protection provided by long-lasting insecticidal nets. PloS One. 2014;9(8):e104967. doi:10.1371/journal.pone.0104967

7. Corbel V, Chabi J, Dabire RK, Etang J, Nwane P, Pigeon O, et al. Field efficacy of a new mosaic long-lasting mosquito net (PermaNet 3.0) against pyrethroid-resistant malaria vectors: a multi centre study in Western and Central Africa. Malar J. 2010;9:113. doi:10.1186/1475-2875-9-113
