# Supplementary material for: Proof-of-concept study for a long-acting formulation of ivermectin injected in cattle as a complementary malaria vector control tool
Source: Parasit Vectors. 2023 Feb 14;16:66. doi: 10.1186/s13071-022-05621-z (PMC9926456; doi:10.1186/s13071-022-05621-z)
Supplement: Supplementary file 2 — Additional file 2. Four-parameter log-logistic model equation. [file 13071_2022_5621_MOESM2_ESM.pdf]

Mortality rate as a function of ivermectin plasma concentration is estimated with à four parameters log logistic function described by the equation below :

$$f(x) = c + \frac{d - c}{1 + \exp(b(\log(x) - \log(e)))}$$

where x is the ivermectin plasma concentration, b the slope, c the lower limit, d the upper limit and e the inflection point, (ED50).
