# Supplementary material for: Proof-of-concept study for a long-acting formulation of ivermectin injected in cattle as a complementary malaria vector control tool
Source: Parasit Vectors. 2023 Feb 14;16:66. doi: 10.1186/s13071-022-05621-z (PMC9926456; doi:10.1186/s13071-022-05621-z)
Supplement: Supplementary file 3 — Additional file 3: Table S3. Hazard ratio value of multiple comparisons. [file 13071_2022_5621_MOESM3_ESM.pdf]

Supplemental Table 3 : Hazard Ratio of multiple comparisons

Comparison	DAI	Hazard Ratio	SE	95%.LCL	95% UCL	z.ratio	p.value
A/B	1	0.9989977	0.5508399	0.2423075	4.118719	-0.001818668	1.000000e+00
A/B	7	0.8226866	0.4535653	0.1995792	3.391201	-0.354021668	9.847915e-01
A/B	14	1.0659128	0.5872348	0.2588505	4.389290	0.115863108	9.994442e-01
A/B	21	0.4207874	0.2327763	0.1015914	1.742884	-1.564786052	3.989008e-01
A/B	28	0.8545543	0.4720943	0.2067130	3.532739	-0.284508353	9.919749e-01
A/B	49	0.5352732	0.2951114	0.1298522	2.206489	-1.133585330	6.687102e-01
A/B	91	0.4992990	0.2751227	0.1212219	2.056556	-1.260485401	5.882033e-01
A/B	105	0.5122422	0.2787195	0.1265888	2.072789	-1.229438153	6.080637e-01
A/B	119	0.9755190	0.5384926	0.2362399	4.028266	-0.044901067	9.999675e-01
A/B	155	1.6480146	0.9086313	0.3997716	6.793760	0.906088944	8.016051e-01
A/B	183	1.5800576	0.8741918	0.3814040	6.545768	0.826838272	8.417690e-01
A /T_A	1	7.2290312	4.0174533	1.7339366	30.138871	3.559414848	2.105219e-03
A /T_A	7	9.9304938	5.5151146	2.3841504	41.362621	4.133466754	2.091723e-04
A /T_A	14	16.0682601	8.9331188	3.8520195	67.026915	4.994793361	3.513642e-06
A /T_A	21	5.0453442	2.7979982	1.2138014	20.971716	2.918414070	1.847158e-02
A /T_A	28	6.1260231	3.4312308	1.4529734	25.828524	3.236068251	6.650673e-03
A /T_A	49	2.7148012	1.5015915	0.6555729	11.242298	1.805632808	2.705841e-01
A /T_A	91	2.5807553	1.4428772	0.6137053	10.852601	1.695756267	3.258551e-01
A /T_A	105	4.0955339	2.2393089	1.0052452	16.685877	2.578599747	4.874348e-02
A /T_A	119	4.2325814	2.3609281	1.0098579	17.739867	2.586618149	4.771102e-02
A /T_A	155	4.1366386	2.2888546	0.9984069	17.139083	2.566150253	5.038370e-02
A /T_A	183	1.8942692	1.0480062	0.4572678	7.847165	1.154689699	6.554891e-01
B /T_B	1	8.4884007	4.7234329	2.0322510	35.454749	3.843422414	7.006068e-04
B /T_B	7	9.6997608	5.3677918	2.3406153	40.196849	4.105755075	2.356466e-04
B /T_B	14	10.0016100	5.5361027	2.4126667	41.461260	4.160177227	1.863370e-04
B /T_B	21	8.5371250	4.7321697	2.0552501	35.461622	3.868673256	6.327735e-04
B /T_B	28	8.5769747	4.8232236	2.0226280	36.370749	3.821638251	7.645415e-04
B /T_B	49	4.8798683	2.6985015	1.1787779	20.201528	2.866468004	2.160342e-02
B /T_B	91	5.9900532	3.4336482	1.3736440	26.120841	3.122858097	9.691624e-03
B /T_B	105	6.5426810	3.6523577	1.5592765	27.452908	3.364792437	4.263042e-03
B /T_B	119	5.1098391	2.9701499	1.1478494	22.747284	2.806257579	2.580765e-02

Comparison	DAI	Hazard Ratio	SE	95%.LCL	95% UCL	z.ratio	p.value
B /T_B	155	3.9760907	2.2627685	0.9215291	17.155505	2.425433490	7.234932e-02
B /T_B	183	1.3911684	0.7965405	0.3195616	6.056264	0.576600685	9.391151e-01
